# *In vitro* membrane protein synthesis inside Sec translocon-reconstituted cell-sized liposomes

**DOI:** 10.1038/srep36466

**Published:** 2016-11-03

**Authors:** Naoki Ohta, Yasuhiko Kato, Hajime Watanabe, Hirotada Mori, Tomoaki Matsuura

**Affiliations:** 1 Department of Biotechnology, Graduate School of Engineering, Osaka University, 2-1 Yamadaoka, Suita, Osaka, Japan; 2 Graduate School of Biological Sciences, Nara Institute of Science and Technology, 8916-5 Takayama-tyou, Ikoma, Nara, Japan.

## Abstract

Protein synthesis using an *in vitro* transcription-translation system (IVTT) inside cell-sized liposomes has become a valuable tool to study the properties of biological systems under cell-mimicking conditions. However, previous liposome systems lacked the machinery for membrane protein translocation. Here, we reconstituted the translocon consisting of SecYEG from *Escherichia coli* inside cell-sized liposomes. The cell-sized liposomes also carry the reconstituted IVTT, thereby providing a cell-mimicking environment for membrane protein synthesis. By using EmrE, a multidrug transporter from *E. coli*, as a model membrane protein, we found that both the amount and activity of EmrE synthesized inside the liposome is increased approximately three-fold by incorporating the Sec translocon. The topological change of EmrE induced by the translocon was also identified. The membrane integration of 6 out of 9 *E. coli* inner membrane proteins that was tested was increased by incorporation of the translocon. By introducing the Sec translocon, the membrane integration efficiency of the membrane protein of interest was increased, and enabled the integration of membrane proteins that otherwise cannot be inserted. In addition, this work represents an essential step toward the construction of an artificial cell through a bottom-up approach.

Membrane proteins, which include transporters, receptors and enzymes, are essential components of cells. Approximately 20% to 25% of open reading frames (ORFs) in the human genome are estimated to encode membrane proteins^1^. In addition, more than 50% of drug targets are membrane proteins^1^,^2^,^3^. Given this biological and pharmaceutical importance, studies of membrane protein have been of great importance^4^,^5^,^6^. However, the hydrophobic nature of membrane proteins makes their *in vitro* characterization difficult. Endogenous expression levels *in vivo* are often low, and overexpression sometimes results in cell toxicity. Furthermore, membrane proteins tend to aggregate during purification due to their hydrophobic nature.

One solution to the aforementioned problems is the use of an *in vitro* transcription and translation system (IVTT), where proteins are synthesized in the test tube without using living cells^7^,^8^,^9^. As IVTT is an open system, membrane protein solubilizing reagents, including lipids, detergents and nanodiscs, can be added to the reaction, thereby enabling the simultaneous synthesis and solubilization of a membrane protein of interest (mPOI)^7^. IVTT can also be encapsulated within cell-sized liposomes (1 μm>)^10^. Cell-sized liposomes can be used to mimic the cellular environment and even to study the effect of confinement^11^ of molecules in a small space^12^,^13^. In addition, cell-sized liposomes can be visualized using flow cytometry (FCM) and/or optical microscopy, which allow the characterization of an mPOI at the single-vesicle level^14^,^15^,^16^. The use of fluorescence-activated cell sorting (FACS) enables the directed evolution of membrane proteins *in vitro*^17^.

To date, various membrane proteins have been synthesized using IVTT inside cell-sized liposomes^10^,^12^,^14^,^15^,^16^,^17^. In pioneering a study, Noireaux *et al.*^10^ demonstrated the synthesis of the pore-forming protein α-hemolysin in its active form inside cell-sized liposomes. Active transporters have also been synthesized inside liposomes. EmrE^18^, a multidrug-transporter from *Escherichia coli*, was synthesized in its active form, exhibiting the transport of substrates across the membrane^12^. Nevertheless, problems remain with this technology. For example, only 20% of EmrE synthesized inside the liposome was integrated into the membrane, and the remaining protein is likely to have aggregated inside the liposome^12^. With α-hemolysin, only 10% of the protein bound to the membrane showed pore-forming activity^13^. Living cells use translocons^19^,^20^, which are essential components of cells, to properly fold and localize membrane proteins in the membrane. Previous methods, however, have relied on spontaneous insertion, without the help of translocons, to achieve membrane protein integration^21^,^22^,^23^,^24^.

In *E. coli*, the emergence of a nascent peptide chain from the ribosome generates a ribosome-nascent chain complex that is first recognized by the signal recognition particle (SRP) and subsequently transported to the SRP receptor (SR) located on the inner membrane surface^25^,^26^. The complexes are then transferred to the SecYEG hetero-trimer complex, where the membrane protein is synthesized, folded and/or localized properly^27^. To date, the Sec translocon has been incorporated into IVTT by the addition of an inverted inner membrane vesicle (INV) prepared from *E. coli* cells^28^,^29^. *In vitro* protein synthesis was performed outside of the INV to investigate the effect and role of the translocon on mPOI synthesis. Alternatively, the SecYEG complex was synthesized using IVTT in the presence of large unilamellar vesicles (LUVs) to generate a functional translocon from the outside of the liposome^30^. However, in all of these previous studies, the mPOI was synthesized outside of the liposome that carries the translocon, yet no studies have reported the synthesis of mPOIs inside of liposomes in which the translocon was reconstituted.

In this study, we aim to reconstitute the translocon consisting of the SRP/SR pathway and SecYEG, which we term here the Sec translocon, inside of cell-sized liposomes along with the membrane protein synthesis machinery. We previously encapsulated reconstituted IVTT, the protein synthesis using recombinant elements (PURE) system^31^, inside cell-sized liposomes composed of defined components and succeeded in engineering^17^,^32^ and characterizing^12^,^13^ the mPOI. However, this technique, termed liposome display, lacked the translocon. By introducing the Sec translocon to the liposome display technique, the membrane integration efficiency of the mPOI is expected to increase. In addition, this strategy should enable the display of membrane proteins that otherwise cannot be inserted. As liposome display provides a platform to study membrane proteins under cell-mimicking conditions and to engineer membrane proteins by directed evolution, introducing the Sec translocon is expected to expand the repertoire of mPOI that are applicable to these studies. In addition, this work represents an essential step toward the construction of an artificial cell through a bottom-up approach^33^,^34^,^35^.

## Results

### *In vitro* batch synthesis of the Sec translocon

In this study, we aimed to reconstitute the Sec translocon consisting of *E. coli* SRP/SR and SecYEG inside liposomes. First, we incorporated all three components, SRP, SR and SecYEG, into the reconstituted IVTT and investigated their effect on batch *in vitro* protein synthesis. SRP is a complex of Ffh and 4.5S RNA, SR is FtsY, and SecYEG is a hetero-trimer of SecY, SecE, and SecG. As Ffh and FtsY were reported to be expressed in soluble form when overexpressed in *E. coli*^36^,^37^, the two proteins were purified and added to the IVTT^28^. Note that 4.5S RNA is included in the tRNA mixture of the IVTT^28^. However, because SecYEG is an integral membrane protein, DNA encoding each subunit of the complex was added to the IVTT. The DNA concentrations of SecY, SecE and SecG were adjusted to achieve equal stoichiometry of the synthesized subunits^27^. Information about the sequence of all proteins used in this study is shown in Supplementary Table S1.

**Figure 1:**
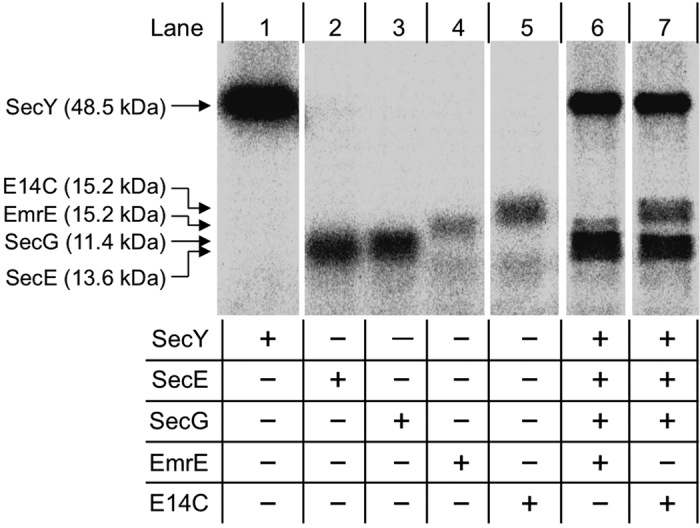
*In vitro* batch protein synthesis of the Sec translocon using the reconstituted IVTT. SecYEG and mPOIs were batch synthesized with the reconstituted IVTT supplemented with [^35^S]-methionine at 37 °C for 2 h. The synthesized proteins were analyzed by autoradiography of the SDS polyacrylamide gel. The concentrations of the DNA encoding EmrE, E14C, SecY, SecE and SecG were 500 pM, 500 pM, 400 pM, 100 pM and 100 pM, respectively. The concentrations of the Ffh and FtsY proteins were 150 nM and 750 nM, respectively.

To investigate the effects of the addition of the Sec translocon to the IVTT on the amount of synthesized mPOI, we analyzed the synthesized proteins by autoradiography of SDS polyacrylamide gels (Fig. 1). EmrE and E14C, a loss-of-function mutant of EmrE^38^, were used as mPOIs. EmrE is an *E. coli* multidrug-transporter that belongs to the small multidrug resistance (SMR) protein family^18^,^39^,^40^. The synthesis of both proteins was detected (Fig. 1, lane 1–5). Then, SecYEG and the mPOIs were synthesized simultaneously (lane 6–7). The molar ratio of EmrE to SecYEG was 1:4.4, and thus the amount of SecYEG was more than that of the mPOI. The amount of SecYEG was calculated as the average of the three subunits. With the addition of the Sec translocon, the amount of synthesized mPOI changed by 0.711 ± 0.180-fold (average value of EmrE and E14C). We thus concluded that the amount of synthesized mPOI was not significantly altered by the incorporation of the Sec translocon.

### The effect of Sec translocon incorporation on EmrE membrane integration

Next, we investigated the effects of the Sec translocon on the integration of EmrE into the cell-sized liposomal membrane. Liposomes were prepared via the water-in-oil (W/O) emulsion transfer method^41^ using 100% 1-palmitoyl-2-oleoyl-*sn*-glycero-3-phosphocholine (POPC). Reconstituted IVTT, DNA encoding the mPOI, and transferrin Alexa Fluor 647 conjugate (TA647) were encapsulated inside the liposome. TA647 was used to estimate the aqueous volume of the liposome, which was found to range from approximately 3 to 400 fL (see the Methods section). EmrE has four transmembrane domains and forms an anti-parallel homodimer^18^, and thus both the N- and C-terminus of one of the two subunits is expected to be outside the liposome. For detection, we fused the Myc-tag to the C-terminus of EmrE and performed immunostaining using Alexa Fluor 488 (AF488)-labeled anti-Myc-tag antibody. The fluorescence intensity of the liposomes was measured using FCM. Note that all FCM data analyses were conducted with liposome populations defined as unilamellar vesicles based on the 2D plot of forward- and side-scattering intensities, as in our previous reports^41^,^42^,^43^, which is approximately 60–70% of the entire plot (Supplementary Fig. S1).

**Figure 2:**
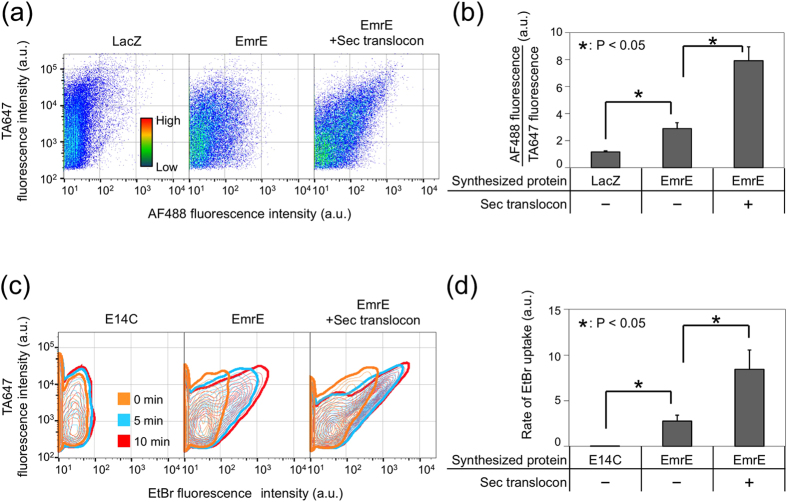
The effect of the Sec translocon on EmrE synthesis inside cell-sized liposomes. (**a**) Two-dimensional FCM data (density plot) of EmrE-displaying liposomes. The vertical axis is TA647 fluorescence intensity, which correlates with the liposome aqueous volume, and the horizontal axis is AF488 fluorescence intensity, which correlates with the amount of membrane-integrated EmrE. LacZ was used as a negative control. The data from 100,000 particles are shown. (**b**) Quantitative analysis of the effect of Sec translocon incorporation on the membrane integration of EmrE. The median AF488/TA647 fluorescence intensity was calculated from the data shown in (**a**). Liposomes showing TA647 fluorescence intensity larger than 10,000 were used. The threshold was used to eliminate liposomes with AF488 fluorescence signal similar or below that of negative control from the quantitative analyses. The average and standard deviation of four independent measurements are shown. The P values (ANOVA) were P = 0.015 and 0.0077 between LacZ and EmrE, and EmrE and Sec translocon-reconstituted EmrE, respectively. (**c**) Two-dimensional FCM data (counter plot) showing the EtBr transport activity of EmrE-displaying liposomes. The vertical axis is TA647 fluorescence intensity, which correlates with the liposome aqueous volume, and the horizontal axis is the EtBr fluorescence intensity of the liposome. The data from 100,000 particles are shown. (**d**) Quantitative analysis of the effect of Sec translocon incorporation on the rate of EtBr uptake. EtBr/TA647 fluorescence was obtained from (**c**), and the median value was calculated for each time point. Then, the slope obtained from linear regression of the plot of time versus the median value was defined as the rate of EtBr uptake. Liposomes showing TA647 fluorescence intensity larger than 10,000 were used. The threshold was used to eliminate liposomes with EtBr fluorescence signal similar or below that of negative control from the quantitative analyses. The average and standard deviation of three independent measurements are shown. The P values (ANOVA) were P = 0.013 and 0.046 between E14C and EmrE, and EmrE and Sec translocon-reconstituted EmrE, respectively. The final concentration of DNA encoding the mPOI (EmrE, E14C, LacZ) was set at 50 pM.

EmrE showed higher AF488 fluorescence than LacZ (β-galactosidase), a soluble enzyme used as a negative control, suggesting that EmrE synthesized inside the liposome was inserted into the liposomal membrane (Fig. 2a, Supplementary Fig. S2a). When the Sec translocon was incorporated, *i.e.*, when *secYEG*-encoding DNAs and SRP/SR proteins were incorporated into the liposomes, the fluorescence signal increase by 2.8 ± 0.33-fold (Fig. 2b). A significant change in the amount of synthesized protein was not observed (Fig. 1); therefore, the results suggest that incorporation of the Sec translocon increased the membrane integration of EmrE.

### The effect of Sec translocon incorporation on the substrate transport activity of EmrE

The increased membrane integration of EmrE shown above does not necessarily reflect an increase in functional EmrE. We therefore investigated the effect of the Sec translocon on the substrate transport activity of EmrE. We used ethidium bromide (EtBr) as an EmrE substrate because of its ability to emit fluorescence once transported into the liposome, given that it binds to the nucleic acids (DNA, rRNA, tRNA, and mRNA) present only inside the liposome.

After EmrE synthesis inside the liposome, EtBr was added to the outside, and EtBr fluorescence inside was detected by FCM^8^,^12^. An increase in EtBr fluorescence was observed over time with EmrE, whereas no increase was detected with E14C, an inactive mutant of EmrE (Fig. 2c). In the presence of the Sec translocon, an even faster fluorescence increase was detected with EmrE (Fig. 2c). The results show that transport activity was increased by 3.3 ± 0.62-fold through the incorporation of the Sec translocon (Fig. 2d, Supplementary Fig. S2b,c). Incorporation of the Sec translocon increased the amount of membrane-integrated and active EmrE by 2.8 ± 0.33- and 3.3 ± 0.62-fold respectively. As both values showed a similar fold increase, the results suggest that the incorporation of the Sec translocon promoted the membrane integration of functional EmrE. The effect of the Sec translocon was further confirmed by omitting SecY, the channel of the translocase^27^,^44^. The removal of SecY alone abolished the effects of the Sec translocon (Supplementary Fig. S3). In addition, SecY showed a positive effect only when SecE and SecG were cosynthesized, whereas SRP/SR showed very little effect (Supplementary Fig. S4).

### Topological analysis of membrane-integrated EmrE

**Figure 3:**
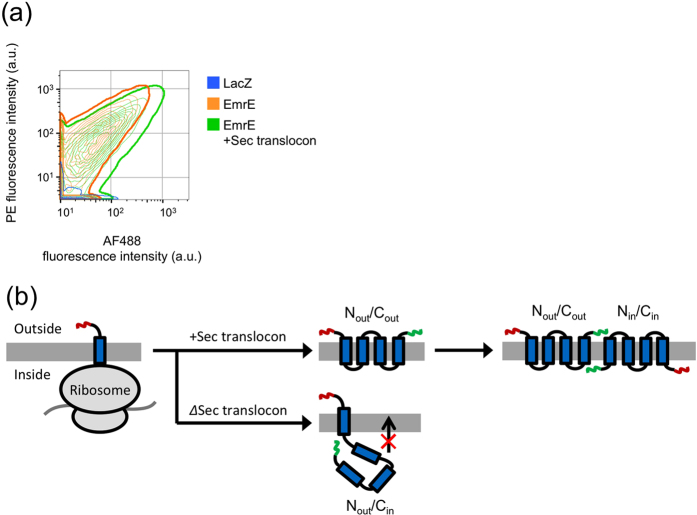
Topological analysis of membrane-integrated EmrE. (**a**) Two-dimensional FCM data (counter plot) of EmrE-displaying liposomes. Immunostaining with both PE-labeled anti-HA-tag antibody and AF488-labeled anti-Myc-tag antibody was simultaneously performed. The vertical axis is the PE fluorescence intensity, and the horizontal axis is AF488 fluorescence intensity. LacZ, a soluble enzyme, was used as a negative control. The final concentration of DNA used was 500 pM. (**b**) Proposed role of Sec translocon on the topology of EmrE. In the absence of Sec translocon, a fraction of EmrE has its N- and C-terminus located outside and inside the liposome, respectively. The figure show only the first transmembrane domain inserted in the membrane as an example but an exact region remains unclear, and other topologies are possible. In the presence of Sec translocon, C-terminus of such EmrE is located outside the liposome with the help of the Sec translocon to form N_out_/C_out_ topology, which then associate with N_in_/C_in_ to form functional antiparallel dimer.

EmrE has four transmembrane domains and forms an antiparallel homodimer. Interestingly, EmrE is known to show dual topology, although the detailed mechanism underlying this topology is not fully understood^45^,^46^. In addition, the role of the translocon has not been investigated. Here, we found that the integration of active EmrE was enhanced by the Sec translocon. To investigate the relationship between the generation of active EmrE and the topological changes caused by the Sec translocon, we generated HA-EmrE-Myc, which incorporates an HA-tag and a Myc-tag at the N- and C-terminus, respectively (Fig. 3). After the synthesis of HA-EmrE-Myc inside the liposome, immunostaining was performed with both Phycoerythrin (PE)-labeled anti-HA-tag antibody and AF488-labeled anti-Myc-tag antibody. The resulting liposomes were analyzed by FCM (Fig. 3a). We observed a shift of the 2D plot only toward increased AF488 fluorescence, indicating that only C-terminal integration was increased by the incorporation of the Sec translocon. These results suggest that the incorporation of the Sec translocon did not increase the amount of integrated EmrE but played a role in exposing the C-terminus to the outside of the liposome, thereby yielding functional EmrE (Fig. 3b). See Discussion section for a possible role of the Sec translocon on the topology of EmrE.

### Effect of Sec translocon incorporation on the synthesis of *E. coli* membrane proteins

**Figure 4:**
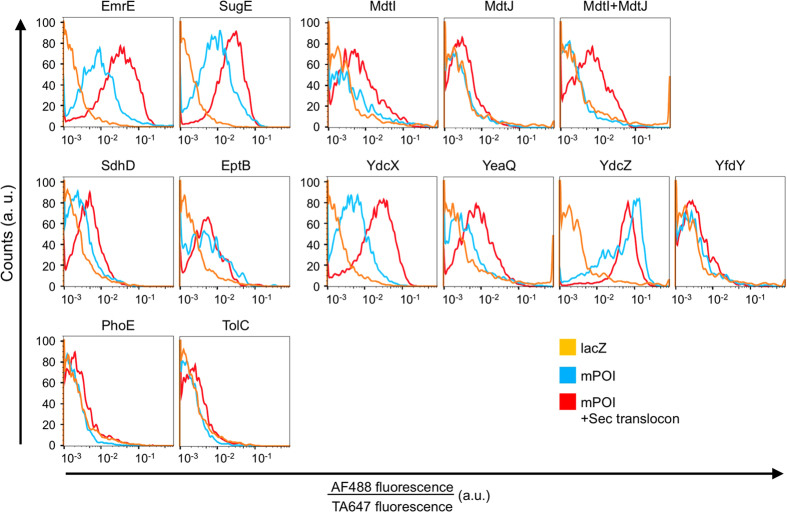
Effect of the Sec translocon on the membrane integration of *E.* *coli* membrane proteins. Effect of the Sec translocon on the membrane integration of 11 mPOIs (9 IMPs and 2 OMPs) from an *E. coli* library are shown. *In vitro* protein synthesis was performed inside cell-sized liposomes at 37 °C for 2 h (EmrE, SugE, YdcX), 4 h (SdhD, EptB, PhoE, TolC), or 16 h (MdtI, MdtI, MdtI/MdtJ, YeaQ, YdcZ, YfdY). The incubation time was extended for clones with weaker fluorescent signals, and thus the incubation time differs among the sequences. Those with 2- or 4-h synthesis were stored overnight at 4 °C. Then, the liposomes were immunostained with AF488-labeled anti-Myc-tag antibody and subjected to FCM analysis. LacZ was used as a negative control. Histograms of AF488/TA647 fluorescence intensity are shown. The top 5 graphs show the results of the SMR family, the middle 6 graphs show the results of IMPs other than SMRs, and the bottom 2 graphs show the results of OMPs. Note that the EmrE shown here is different from that in Fig.2. EmrE here has Myc-tag at both N- and C-terminus, while the one in Fig. 2 has the tag only at the C-terminus. The concentration of DNA encoding each mPOI was 50 pM.

We next investigated the effect of incorporating the Sec translocon on membrane proteins other than EmrE (Fig. 4). As mPOIs, eight inner membrane proteins (IMPs) with 2 to 5 transmembrane domains (Supplementary Table S1), as well as two outer membrane proteins (OMPs), were selected, and the encoding DNA was obtained from an *E. coli* K-12 ORF library^47^. The chosen IMPs can be classified into three groups: (1) the SMR family^40^,^48^, which includes EmrE, (2) IMPs other than the SMR family whose function is known, and (3) IMPs that belong to the Y gene whose detailed functions remain unknown. For detection purposes, Myc-tags were fused to both the N- and C-termini of each sequence. After the synthesis of each membrane protein, the liposomes were immunostained with AF488-labeled anti-Myc-tag antibody and analyzed by FCM^40^,^48^.

The results are shown in Fig. 4 and in Supplementary Fig. S5 online. Two proteins of the SMR family, EmrE and SugE, both of which form homodimers and exhibit dual topology, were integrated into the liposomal membrane by spontaneous insertion. In addition, the amount of membrane insertion was increased by 3.68- and 2.66-fold, respectively, by the incorporation of the Sec translocon. Note that the EmrE shown in Fig. 4 has Myc-tag at both N- and C-terminus, while the one shown in Fig. 2 has the tag only at the C-terminus. We also evaluated a heterodimeric SMR protein, MdtI/MdtJ^40^. While the integration of MdtI, MdtJ, or MdtI/MdtJ was hardly detectable without the Sec translocon, incorporation of the translocon increased the fluorescence of only MdtI/MdtJ (3.23-fold) but not of MdtI or MdtJ. Detection of the membrane integration of MdtI/MdtJ was possible only when the Sec translocon was present. MdtI and MdtJ are known to form a heterodimer^40^. We thus postulate that MdtI or MdtJ alone cannot get integrated into the membrane properly, whereas when both MdtI and MdtJ are present, topology of one or both of the proteins are affected by the Sec translocon, resulting in an assembly of a heterodimer that can get integrated into the membrane. With two other IMPs tested (SdhD and EptB), integration of SdhD was increased by 1.91-fold, whereas EptB showed little change by the incorporation of the Sec translocon. With the four Y genes we tested (YdcX, YeaQ, YdcZ and YfdY), the integration of YdcX and YeaQ was increased by 4.82- and 2.21-fold, respectively, whereas the other two proteins showed very little effect. Finally, the integration of both OMPs (PhoE and TolC) could not be detected regardless of the presence of the Sec translocon.

From these results, the membrane integration of 6 out of 9 IMPs tested (Fig. 4, Supplementary Fig. S5) was increased by more than approximately two-fold by the incorporation of the Sec translocon. All of the proteins that showed an increase were IMPs, and no detectable changes were observed with OMPs. To expand the repertoire of this system, further modification of the liposome display system by incorporating SecA and SecB^49^ and/or altering the phospholipid composition^50^,^51^,^52^,^53^ to mimic that of *E. coli* may be necessary.

## Discussion

We reconstituted the Sec translocon inside of cell-sized liposomes, which resulted in an increase in the production of active EmrE by approximately three-fold. While we have not analyzed the complex formation of SecY, SecE, and SecG, increase in the active EmrE suggest the formation of complexes. In addition, the membrane integration of 6 out of 9 *E. coli* IMPs was increased. In particular, heterodimeric transporter MdtI/MdtJ was detected only in the presence of the Sec translocon. These results suggest that introducing the Sec translocon expanded the repertoire of membrane proteins that are applicable to studies using cell-sized liposomes. In addition, this work represents an essential step toward the construction of an artificial cell using a bottom-up approach.

Reconstituting the membrane integration processes *in vitro* has been previously achieved^28^,^29^,^30^,^54^. With these reconstituted systems, liposomes that are smaller than 1 μm (*i.e.,* LUVs) or INVs carrying SecYEG were used to solubilize the synthesized mPOIs, and membrane integration was analyzed mostly by protease protection assays. In the present study, we reconstituted the Sec translocon using giant unilamellar vesicles (GUVs), a more cell-like environment, and the mPOI was visualized by immunostaining with fluorescently labeled antibody and FCM. Immunostaining enabled the analysis of the various membrane proteins under the same experimental set up, as long as antibodies that bind to the extra-vesicular region are available^12^,^32^. Furthermore, as the cell-sized liposome can be used for directed evolution of membrane proteins^17^, a wide range of mPOIs, including pharmaceutically important targets such as G-protein-coupled receptors and transporters, which might require the translocon for their membrane integration, may become possible.

The homodimeric multidrug transporter EmrE shows dual topology. This topology requires two identical sequences to be inserted in different orientations, which is very intriguing^55^,^56^. As EmrE is known to have four transmembrane domains, both the N- and the C-terminus of one subunit are located on the same side of the membrane^57^. When both termini face the cytosol or periplasm, the topology is termed N_in_/C_in_ or N_out_/C_out_ (Fig. 3b, right figure)^46^. Two mechanisms have been proposed to explain this dual topology^46^. The co-translational model assumes that both topologies (N_in_/C_in_ or N_out_/C_out_) are generated in equal proportions during the membrane integration process and that the topology does not change afterwards. Conversely, the post-translational model assumes the occurrence of a flip-flop of the monomer after membrane integration until the antiparallel dimer is formed. Although such mechanisms have been discussed, the role of the translocon on topology determination remains unclear^56^. Our results suggest that the N_out_/C_in_ domain was converted to N_out_/C_out_ by the incorporation of the translocon (Fig. 3b). Flipping of the N_in_/C_in_ to N_out_/C_out_, and vice versa is unlikely to be promoted by the translocon, as such a function should affect the fluorescence signal from both termini, which was not the case (Fig. 3). Although they need to be confirmed *in vivo*, our results suggest how the translocon contributes to the generation of the dual topology: polypeptides with N_out_ have a topology of N_out_/C_in_ rather than N_out_/C_out_, but the former is converted to the later by the translocon. However, the generation of N_in_/C_in_ is largely unaffected by the translocon.

Detecting the terminal tag-sequences by fluorescence labeled antibodies enabled the analysis of membrane integration of various mPOIs using the same experimental set up, however, the method also has some limitations. The presence of tag-sequences may affect the membrane integration processes of mPOIs. In particular, the effect might be significant when the size of the mPOI sequence is similar to that of tag-sequences. In addition, analyzing terminal tag-sequences alone cannot distinguish whether the detected increase in the fluorescence signal is due to topological changes of mPOIs, as with EmrE, or increased membrane integration. Additional analysis including protease protection assay^28^,^30^ or use of sequence or conformational specific antibodies^58^ are required to clarify the details.

The constructed system differs from *E. coli* cells in several ways. In *E. coli*, proteins of interest are synthesized when SecYEG is present, whereas we co-synthesized both the mPOI and SecYEG. Our system also lacks the SecA and SecB, which are necessary for membrane translocation. We used only phosphatidylcholine as a phospholipid, whereas the *E. coli* membrane consist of other phospholipids. Phospholipid composition has been reported to play an important role in determining the topology of membrane proteins^58^. Despite these differences, we have shown the efficacy of incorporating the Sec translocon in displaying various membrane proteins. Nevertheless, step-by-step improvement of the system is essential not only for various applications using cell-sized liposomes but also for artificial cell assembly using bottom-up approaches^33^,^34^,^35^.

## Methods

### Preparation of PCR products

PCR products were used as templates for *in vitro* protein synthesis. PCR products were prepared using plasmid DNA as a template via two-step PCR amplification using the primers listed in Supplementary Tables S2 and S3 online. The first PCR was performed using KOD-Plus- (Toyobo, Osaka, Japan) or Herculase II fusion DNA polymerase (Agilent Technologies, Tokyo, Japan), and the second PCR was performed using KOD-Plus-. The PCR product was purified using a QIAquick PCR purification kit (Qiagen, Hilden, Germany) according to manufacturer’s instructions. The plasmids used as a template were pET-EmrE-myc^12^, encoding EmrE, pET-E14C-myc^12^, encoding the E14C mutant of EmrE, and pBAD22-hisEYG, encoding SecYEG. Plasmids encoding the 12 membrane proteins shown in Fig. 4 were obtained from the ASKA library (NBRP, Shizuoka, Japan). Information about the sequence of all proteins used in this study is shown in Supplementary Table S1.

### *In vitro* batch protein synthesis using reconstituted IVTT

The IVTT used in this study was a reconstituted *in vitro* translation system (the PURE system) prepared in the laboratory^59^,^60^. When reconstituting the Sec translocon, 150 nM Ffh and 750 nM FtsY were added. When necessary, the synthesized proteins were labeled with [^35^S]methionine and analyzed via SDS-PAGE and autoradiography. Protein bands were detected on a Typhoon FLA 7000 biomolecular imager (GE Healthcare UK Ltd., Buckinghamshire, England).

### *In vitro* protein synthesis inside cell-sized liposomes

Liposomes containing the reconstituted IVTT were prepared using the W/O emulsion transfer method^41^,^42^ essentially as described in our previous report^43^. The liposomal membrane was constructed only with POPC (Avanti Polar Lipids, Alabaster, AL). POPC was dissolved in chloroform at a concentration of 100 mg/mL. Liquid paraffin (Wako Pure Chemical Industries, Osaka, Japan) was then added to bring the lipid concentration to 5 mg/mL, and the samples were heated at 80 °C for 1 h to completely dissolve the lipids and evaporate the chloroform. The product was designated as the oil phase. Next, 20 μL of the PURE system supplemented with the template DNA, 150 nM Ffh, 750 nM FtsY, 2 μM ribosome, 200 mM sucrose, 0.8 U/μL of RNase inhibitor (RNasin Plus; Promega, Madison, WI), and 1.5 μM TA647 (Life Technologies, Carlsbad, CA), was added to 200 μL of the oil phase. TA647 was used to estimate the aqueous volume of each liposome by FCM. The mixtures were vortexed for 30 s to form W/O emulsions that were then equilibrated on ice for 10 min. An aliquot of 200 μL of this solution was gently placed on top of 200 μL of the outer solution containing the low-molecular-weight components of the PURE system (0.357 mM of 18 amino acids, 0.3 mM tyrosine and cysteine, 3.75 mM ATP, 2.5 mM GTP, 1.25 mM CTP and UTP, 1.5 mM spermidine, 25 mM creatine phosphate, 1.5 mM dithiothreitol, 0.01 μg/μL 10-formyl-5,6,7,8-tetrahydrofolic acid, 280 mM potassium glutamate, 19 mM magnesium acetate, and 100 mM HEPES-KOH (pH 7.6)) supplemented with 200 mM glucose and was centrifuged at 18,000 × *g* for 20 min at 4 °C. The pelleted liposomes were collected through an opening at the bottom of the tube. Protein synthesis inside the liposome was conducted at 37 °C for 2 h, 4 h, or overnight after the addition of 0.025 U/μL DNase I (Takara Bio Inc., Shiga, Japan).

### FCM analysis

To detect the Myc-tag or HA-tag located at the termini of the membrane proteins displayed on the liposome surface, AF488-labeled anti-Myc-tag antibody (MBL, Aichi, Japan) or PE-labeled anti-HA-tag antibody (Abcam, Cambridge, UK) was added (final concentration of 5 μg/mL) to the liposome suspension and incubated at 37 °C for 30 min. The unbound antibody was removed by first diluting the liposome 5-fold with dilution buffer (100 mM HEPES-KOH (pH 7.6), 280 mM potassium glutamate, 19 mM magnesium acetate, and 200 mM glucose), centrifuging at 6,000 × *g* for 5 min at 4 °C, discarding the supernatant, and resuspending the liposomes with dilution buffer. The resulting liposomes were analyzed using FCM. To detect the uptake of EtBr, the external solution of the liposome was replaced with dilution buffer containing 0.5 μg/mL of EtBr at pH 8.1.

The fluorescent signals from AF488, PE, EtBr, and TA647 were measured using FCM (FACSVerse; BD Bioscience, Franklin Lakes, NJ) (Single color compensation controls are shown in Supplementary Fig. S6). The total fluorescence intensity of 100,000 liposomes was measured and subjected to analysis. All quantitative analyses were conducted with liposome populations defined as unilamellar liposomes based on the 2D plot of forward- and side-scattering intensities, as in our previous report^43^, which is approximately 60–70% of the entire plot (Supplementary Fig. S1). For quantitative analyses, the median fluorescence intensity of liposomes whose TA647 fluorescence intensity was larger than 10,000 was used. The threshold was used to eliminate liposomes with AF488 or EtBr fluorescence signal similar or below that of negative control from the quantitative analyses. The fraction used for analyses was approximately 10 to 25% of the population. The volume was calculated using equation (1), where *v* (fL), *FI*_TA647_ and *C*_TA647_ (M) are the volume of the liposome, the TA647 fluorescence intensity and the TA647 concentration, respectively.


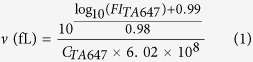


The equation was obtained by measuring the fluorescence of Quantum Alexa Fluor 647 MESF (Bands Laboratories, USA) as a standard with FCM. AF488, PE, and EtBr were excited with a 488-nm semiconductor laser, and the emission was detected through 530 ± 15 nm (AF488) and 616 ± 11 nm (PE, EtBr) band-pass filters. TA647 was excited with a HeNe laser (633 nm), and the emission was detected through a 660 ± 10 nm band-pass filter.

## Additional Information

**How to cite this article**: Ohta, N. *et al.* *In vitro* membrane protein synthesis inside Sec translocon-reconstituted cell-sized liposomes. *Sci. Rep.* **6**, 36466; doi: 10.1038/srep36466 (2016).

**Publisher’s note:** Springer Nature remains neutral with regard to jurisdictional claims in published maps and institutional affiliations.

## Supplementary Material

Supplementary Information
