# Supplementary material for: In vitro membrane protein synthesis inside Sec translocon-reconstituted cell-sized liposomes
Source: Sci Rep. 2016 Nov 3;6:36466. doi: 10.1038/srep36466 (PMC5093552; doi:10.1038/srep36466)
Supplement: Supplementary Information [file srep36466-s1.pdf]

***In vitro* membrane protein synthesis inside Sec translocon-reconstituted cell-sized liposomes**

Naoki Ohta[1], Yasuhiko Kato[1], Hajime Watanabe[1], Hirotada Mori[2], and Tomoaki Matsuura[1]*

[1]Department of Biotechnology, Graduate School of Engineering, Osaka University
[2]Graduate School of Biological Sciences, Nara Institute of Science and Technology

Supplementary Table S1. List of *E. coli* membrane proteins used in this study.

Gene name	Length in amino acids	Number of transmembrane domains[1]	Protein ID[2]	Function of Product[2]
emrE	110	4	1786755	Multidrug resistance protein
sugE	105	4	87082378	Multidrug efflux system protein
mdtI	109	4	1787883	Multidrug efflux system transporter
mdtJ	121	4	1787884	Multidrug efflux system transporter
sdhD	115	3	1786941	Succinate dehydrogenase
eptB	563	5	87082286	KDO phosphoethanolamine transferase, Ca^{2+} inducible
ydcX	57	2	87081912	Conserved protein
yeaQ	82	3	1788096	Conserved protein
ydcZ	149	5	1787718	Inner membrane protein
yfdY	80	3	1788720	Putative inner membrane protein
phoE	351	-	1786436	Outer membrane phosphoporin protein E
tolC	493	-	87082199	Transport channel
secY	433	10	1789696	Preprotein translocase membrane subunit
secE	127	3	1790413	Preprotein translocase membrane subunit
secG	110	2	1789565	Preprotein translocase membrane subunit
ffh	453	-	1788963	Signal recognition particle (SRP) component with 4.5S RNA
ftsy	497	-	1789874	Signal recognition particle (SRP) receptor

1. Number of transmembrane domain was calculated using *TMHMM Server v. 2.0*. Available at: http://www.cbs.dtu.dk/services/TMHMM/. (Accessed: 12th Jun 2015)
2. The function of product indicated is obtained from database: *PEC (Profiling of E. coli Chromosome) Ver.4*, available at: https://shigen.nig.ac.jp/ecoli/pec/, and *E. coli database collection (ECDC)*, available at: http://www.uni-giessen.de/~gx1052/ECDC/ecdc.htm. (Accessed: 20th May 2016)

Supplementary Table S2. Sequence of the primers used in this study.

Primer	Sequence
P1	5'-CTTTAAGAAGGAGATATACCAATGAACCCTTATATTTATCTTGG-3'
P2	5'-GCTAGTTATTGCTCAGCGG-3'
P3	5'-GAAATTAATACGACTCACTATAGGGAGACCACAACGGTTTCCCTCTAGAAATAATTTTGTTTAACTTTAAGAAGGAGATATACCA-3'
P4	5'-CTTTAAGAAGGAGATATACCAATGGCTAAACAACCGGGATTAG-3'
P5	5'-ATACATGAATGGATCCTTATCGGCCGTAGCCTTTCAGG-3'
P6	5'-CTTTAAGAAGGAGATATACCAATGAGTGCGAATACCGAAGCTC-3'
P7	5'-ATTTCCAAGGTTAGAACCTCAGGCCAGTGATAAAG-3'
P8	5'-CTTTAAGAAGGAGATATACCAATGTATGAAGCTCTTTTAGTAG-3'
P9	5'-ATGCATGCCGCGGATCCTTAGTTCGGGATATCGCTGGTCG-3'
P10	5'-CGCGGATCCGTAATACGACTCACTATAGGGTCTAGAGTTTAACTTTAAGAAGGAGATATACATATGTACCCATACGATGTTCCAGATTACGCTACGGATCCGGCCCTGAGGG-3'
P11	5'-TTACAAATCTTCTTCACTTATTAATTTTTGCTCAACATATGCCTCAAATTCACTGCGGCCGCATAGGCC-3'
P12	5'-TAACTTTAAGAAGGAGATATACCATGGAACAGAAACTGATTAGCGAAGAAGATCTGCCGGCCCTGAGGGCC-3'
P13	5'-TACTGCGGCCGCATAGGCC-3'
P14	5'-TTAATACGACTCACTATAGGGAGACCACAACGGTTTCCCTCTAGAAATAATTTTGTTTAACTTTAAGAAGGAGATATACCATG-3'
P15	5'-TTATTACAGATCTTCTTCGCTAATCAGTTTCTGTTCACTGCGGCCGCATAGGCC-3'

Supplementary Table S3. Primer sets used for PCR amplification of each gene.

Template plasmid	Amplified sequence		Forward primer	Reverse primer
pET-EmrE-myc	EmrE-Myc	1st	P1	P2
		2nd	P3	P2
pET-E14C-myc	E14C-Myc	1st	P1	P2
		2nd	P3	P2
pBAD22-hisEYG	SecY	1st	P4	P5
		2nd	P3	P5
	SecE	1st	P6	P7
		2nd	P3	P7
	SecG	1st	P8	P9
		2nd	P3	P9
pCA24N	HA-EmrE-Myc[1]		P10	P11
from ASKA library	Myc-ORF-Myc[2]	1st	P12	P13
		2nd	P14	P15

1. HA-tag or Myc-tag was added to the N- or C-terminal of EmrE sequence, respectively.
2. Myc-tag was added to both N- and C-terminal of mPOI sequence.

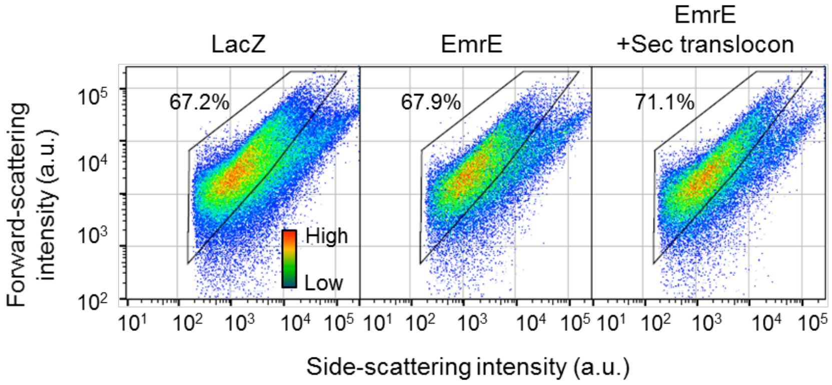

Supplementary Figure S1. Population of liposome used for analysis.

Two-dimensional FCM data (density plot) of side- or forward-scattering intensity of EmrE-displaying liposomes. The data of the samples depicted in Fig. 2 are shown. The gated region indicates liposomes used for data analyses. Results of 100,000 particles are shown. Approximately 60-70% of data in the defined gate and used for analysis.

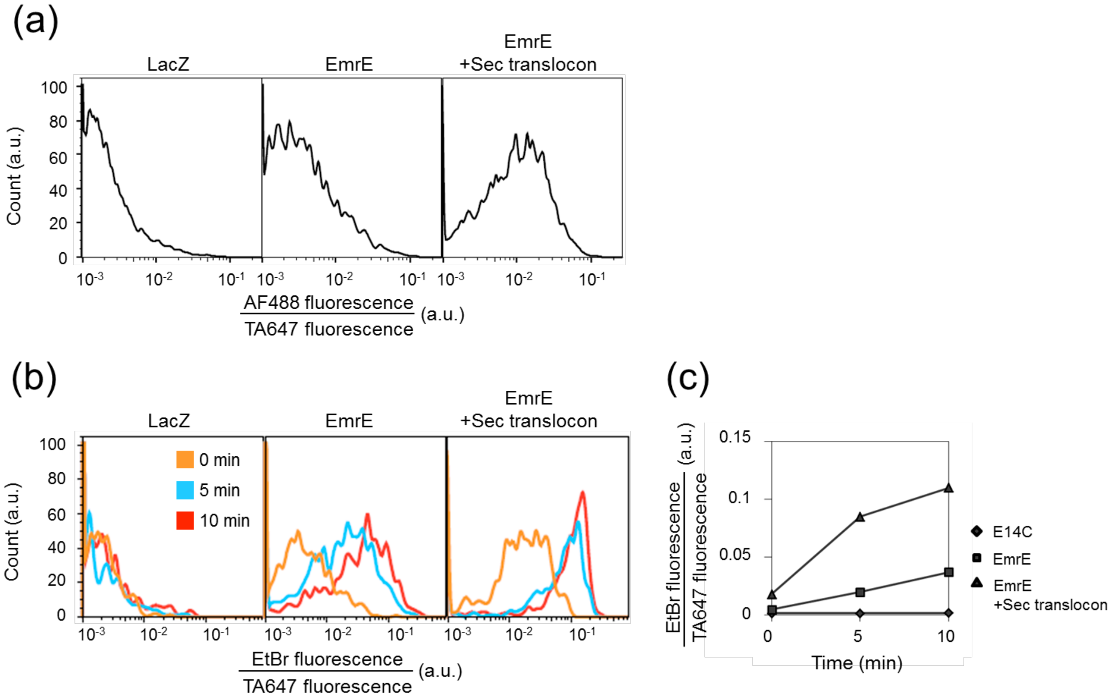

Supplementary Figure S2. Modified data of Fig. 2a and 2c.

(a) The histogram data of AF488/TA647 fluorescence intensity of EmrE-displaying liposomes obtained from the FCM data shown in Fig.2a. Liposomes with TA647 fluorescence larger than 10,000 were used. The average and standard deviation of calculated median from four independent measurements are shown in Fig. 2b. (b) The histogram data of EtBr/TA647 fluorescence intensity of EmrE-displaying liposomes obtained from Fig. 2c. The time-dependent changes in EtBr uptake are shown by each color. (c) The time-dependent changes of median calculated from (b) are shown. The rate from 0 min to 5 min was defined as the rate of EtBr uptake. The average and standard deviation from three independent measurements are shown in Fig. 2d.

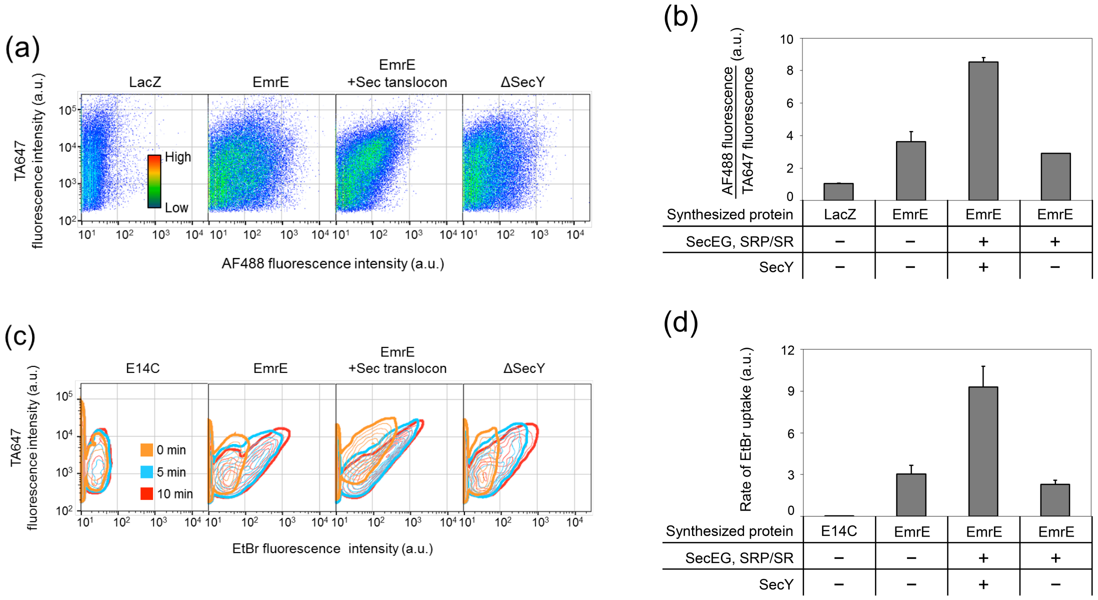

Supplementary Figure S3. The effect of the removal of SecY from the Sec translocon.

(a) Two-dimensional FCM data (density plot) of EmrE-displaying liposomes. The vertical axis is TA647 fluorescence intensity, which correlates with the liposome aqueous volume, and the horizontal axis is AF488 fluorescence intensity, which correlates with the amount of membrane-integrated EmrE. LacZ was used as a negative control. The removal of only the SecY from the Sec translocon is indicated as ΔSecY. The date from 100,000 particles are shown. (b) Quantitative analysis of the effect of Sec translocon incorporation on the membrane integration of EmrE. The median AF488/TA647 fluorescence intensity was calculated from the data shown in (a). The average and standard deviation of two independent measurements are shown. (c) Two-dimensional FCM data (counter plot) showing the EtBr transport activity of EmrE-displaying liposomes. The vertical axis is TA647 fluorescence intensity, which correlates with the liposome aqueous volume, and the horizontal axis is the EtBr fluorescence intensity of the liposome. The time-dependent change in EtBr uptake is shown by each color. The removal of the SecY alone from the Sec translocon is shown as ΔSecY. The data from 100,000 particles are shown. (d) Quantitative analysis of the effect of Sec translocon incorporation on the rate of EtBr uptake. EtBr/TA647 fluorescence was obtained from (c), and the median value was calculated for each time point. Then, the slope obtained from linear regression of the plot of time versus the median value was defined as the rate of EtBr uptake. The average and standard deviation of two independent measurements are shown. The final concentration of the DNA encoding the mPOI (EmrE, E14C, LacZ) was set at 50 pM.

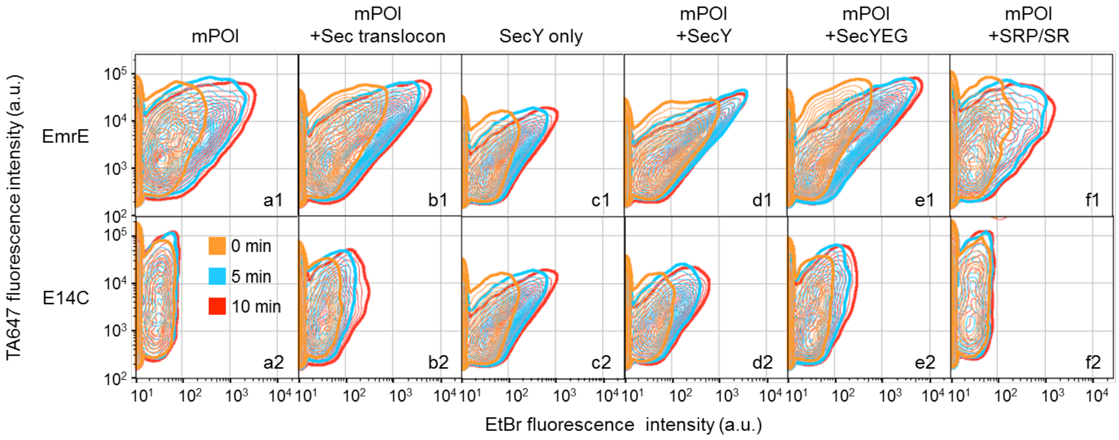

Supplementary Figure S4. Contribution of the components constituting the Sec translocon.

Two-dimensional FCM data (counter plot) of the EtBr transport activity of EmrE- or E14C-displaying liposomes. The vertical axis is TA647 fluorescence intensity, which correlated with the liposome aqueous volume, and the horizontal axis is EtBr fluorescence intensity of the liposome. The time-dependent change in EtBr uptake is shown by each color. E14C was used as negative control of each condition. The data from 100,000 particles are shown. In Supplementary Fig. S3, we show that removal of SecY alone abolished the positive effect of the Sec translocon. We here investigated if SecY alone can exhibit the effect observed with the Sec translocon. When SecY alone was synthesized inside liposomes, EtBr influx was observed (c1,c2). The influx was observed regardless of the presence of mPOIs (d1, d2). These results indicate that SecY is likely to have disrupted the membrane structure and enabled the penetration of EtBr. On the other hand, when SecY was synthesized together with SecE and SecG, EtBr influx was suppressed with E14C, while the flux was detected with EmrE (e1, e2). These results indicate that SecE and SecG have associated with SecY, and the complex did not disrupt the membrane structure. Lastly, the effect of adding only the SRP/SR to the synthesis of mPOI was investigated (f1, f2). As these results were similar to that without SRP/SR (a1, a2), SRP/SR is likely to have very little effect on EmrE membrane integration.

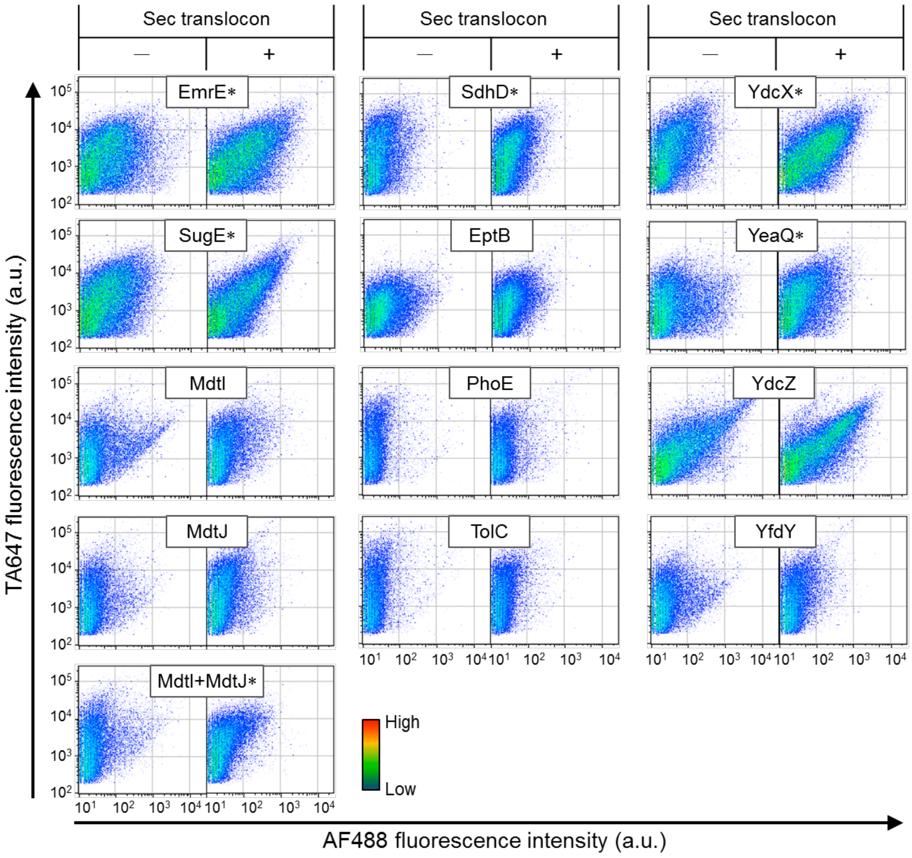

Supplementary Figure S5. Effect of incorporating the Sec translocon on the synthesis of *E. coli* membrane proteins.

Two-dimensional FCM data (density pot) of mPOI-displaying liposomes. The vertical axis is TA647 fluorescence intensity, which correlates with the liposome aqueous volume, and the horizontal axis is AF488 fluorescence intensity, which correlates with the amount of membrane-integrated mPOI. These are the data used to obtain the plots shown in Fig. 4. mPOIs that showed more than approximately two-fold increase by the addition of the Sec translocon are indicated by *.

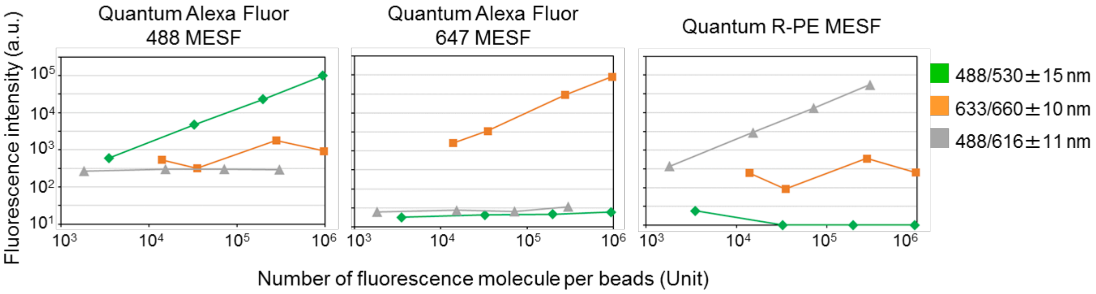

Supplementary Figure S6. Compensation controls.

The fluorescence intensities of control fluorescent beads measured by FCM are shown. Three beads were used: Quantum Alexa Fluor 488 MESF, Quantum Alexa Fluor 647 MESF, Quantum R-PE MESF (Bands Laboratories, USA). All beads were measured with three different excitation/emission wavelengths (488/530±15 nm (AF488), 633/660±10 nm (TA647), 488/616±11 nm (PE, EtBr)). The results show that compensation is done properly.
